# Distribution and Genome Epidemiological Characteristics of *Salmonella* Isolates in Low Water Activity Foods

**DOI:** 10.3390/foods15183248

**Published:** 2026-09-14

**Authors:** Fen Zhou, Meiyi Xu, Xingdi Liu, Yunhe An, Jie Deng, Xiaohui Lin, Wei Wang, Qiushui Wang

**Affiliations:** 1 College of Food Science and Biotechnology, Tianjin Agricultural University, Tianjin 300392, China; zzhoufen@163.com (F.Z.);; 2 Tianjin Institute of Gynecology and Obstetrics, Tianjin Central Hospital of Gynecology and Obstetrics, Tianjin 300100, China; 3 Institute of Analysis and Testing, Beijing Academy of Science and Technology (Beijing Center for Physical and Chemical Analysis), Beijing 100089, China; anyunhe_bio@163.com (Y.A.);; 4 China National Center for Food Safety Risk Assessment, Beijing 100022, China

**Keywords:** *Salmonella*, low-water-activity food, whole-genome sequencing, antimicrobial resistance, *mcr-9.2*, IncHI2 plasmid

## Abstract

*Salmonella* remains a leading global foodborne pathogen, whereas low-water-activity foods are often underestimated as transmission vehicles. Here, whole-genome sequencing and bioinformatic analyses were performed on 59 *Salmonella* strains isolated from low-water-activity foods (2017–2025) to investigate their genomic traits, antimicrobial resistance profiles, virulence genes, and mobile genetic elements. The isolates were mainly recovered from dried meat (44.07%) and dried soybean products (33.90%), covering 33 serovars and 36 sequence types. *Salmonella* Agona (ST13), *S*. Senftenberg (ST14), and *S*. Idikan (ST1561) predominated. Forty antimicrobial resistance genes across ten categories were detected. Notably, one *S*. Schwarzengrund isolate harbored the IncHI2 plasmid pWW013_mcr-9.2 carrying the *mcr-9.2* gene, as well as various antibiotic resistance genes and heavy metal resistance operons. Comparative genomics revealed a conserved plasmid backbone and a distinctive resistance island. This study documents the first detection of an *mcr-9.2*-carrying *Salmonella* in low-water-activity foods in Beijing. Our findings demonstrate high genomic diversity, widespread resistance determinants, and the emergence of an *mcr-9.2*-carrying plasmid, supplying key genomic evidence for risk assessment and targeted control of *Salmonella* in low-water-activity foods.

## 1. Introduction

*Salmonella* constitutes one of the primary agents responsible for foodborne gastroenteritis across the globe. It creates considerable public-health risks and brings heavy economic losses to both health-care infrastructures and the food sector [1]. It is estimated that non-typhoidal salmonellosis is responsible for approximately 93.8 million *Salmonella* infection cases and 155,000 deaths worldwide yearly [2]. Gastroenteric *Salmonella* infections usually develop as self-limiting gastroenteritis, which can progress to bacteraemia and meningitis, especially in immunocompromised patients or those at the extremes of age such as infants (<12 months) and the elderly (≥60 years) [3]. *Salmonella* exhibits virulence factors that play a decisive role in systemic infections, such as pathogenicity islands (PAIs), invasion and adherence genes, and enterotoxin genes [4,5]. In addition, the global emergence of multi-drug-resistant (MDR) *Salmonella* has exacerbated treatment options. This situation is particularly concerning given the spread of plasmid-mediated β-lactam and cephalosporin resistance genes, which pose additional challenges to clinical treatment [6,7].

Over the preceding decade, the global emergence and rapid dissemination of mobile phosphoethanolamine transferase *mcr* genes, responsible for transferable colistin resistance in *Enterobacterales*, has underscored the escalating global public health challenge of antimicrobial resistance (AMR) [8,9]. Following the first description in 2015, several reports describing the presence of *mcr-1* in different host species and geographic areas were published [10,11]. Thus far, the *mcr* gene family comprises *mcr-1* to *mcr-11*, among which *mcr-1* and *mcr-9* are the most widely disseminated [12,13,14]. While the *mcr-1* gene is mobilizable via the composite transposon Tn*6330*, *mcr-9* variants occur within highly variable genomic contexts. These genes can be flanked by IS*1*- or IS*6*-family insertion sequences, or alternatively integrated into class 1 integrons; their transcriptional activity is frequently governed by the *qseC/qseB* regulatory system [15,16,17,18]. This inducibility complicates phenotypic surveillance, as *mcr-9* carriers can appear colistin-susceptible under standard laboratory conditions. Additional complexity arises from neighboring MGEs that restructure the genomic context surrounding *mcr-9*. This complexity renders the association of *mcr-9* with IncHI2/IncHI2A plasmids particularly concerning, as these elements act as insidious drivers of AMR dissemination across clinical, veterinary, and environmental settings.

Most cases of *Salmonella* infection are caused by the consumption of contaminated foods, including raw or undercooked meats, eggs, raw milk, and contaminated water [19]. Foods with low-water-activity are traditionally considered microbiologically safe products due to their inhibitory environment for microbial growth. In recent years, however, accumulating evidence indicates that *Salmonella* can survive in these matrices for extended periods, such as dehydrated food powders/spices, infant formula, wheat flour, vegetable seeds, and tree nuts, serving as a persistent reservoir for human infection [20,21,22,23,24,25]. *Salmonella* is known to exhibit increased thermal resistance in low-water-activity foods and can persist for up to 2 years in these foods [26]. The unique stress conditions in low-water-activity foods may further drive the transmission of MDR strains along the food supply chain. Despite this, genomic studies focusing specifically on *Salmonella* isolated from low-water-activity foods are relatively scarce compared to those from high-moisture foods (e.g., meat, dairy).

In this study, we reported the isolation and identification of *Salmonella* strains from low-water-activity foods in Beijing. The phylogenetic relationships, antimicrobial resistance and virulence genes, and their associated MGEs, especially the characteristics of the emergence of the *mcr-9.2*-carrying isolate, were also elucidated. This work aims to provide critical genomic insights for risk assessment and the development of targeted control strategies against *Salmonella* in low-water-activity foods.

## 2. Materials and Methods

### 2.1. Strain Collection and Whole-Genome Sequencing

Low-water-activity food samples with a minimum weight of 250 g were obtained from various sampling sites throughout Beijing. All specimens were sealed in sterile airtight bags with biological ice packs and kept under refrigeration below 4 °C during transportation, and microbiological processing was initiated within 2 h after sample collection. The isolation of *Salmonella* spp. relied on enrichment and selective culture procedures described in the Chinese national food safety standard GB 4789.4, where relevant culture media and incubation conditions for foodborne *Salmonella* are specified.

A total of 59 *Salmonella* strains from low-water-activity foods in Beijing, China, were collected from 2017 to 2025 in this study (Supplementary Table S1). In particular, a special monitoring program was conducted in 2025 to collect *Salmonella* from low-water-activity foods. Prior to whole-genome sequencing, these isolates were validated as *Salmonella* spp. via invA-targeted PCR [27], and no PCR-dependent screening for antimicrobial resistance determinants was carried out ahead of sample sequencing. Genomic DNA of the above 59 strains was extracted and purified using an Omega EZNA Bacterial DNA kit (Omega Bio-Tek, Norcross, GA, USA) according to the manufacturer’s procedures. The harvested DNA was qualified by agarose gel electrophoresis and quantified by a Qubit 2.0 fluorometer (Thermo Fisher Scientific, Waltham, MA, USA). For each *Salmonella* isolate, genomic DNA was mechanically fragmented by sonication to yield 350 bp fragments. After end-polishing and A-tailing procedures, full-length sequencing adapters were ligated to the DNA termini, and adapter-ligated fragments were enriched by PCR. AMPure XP beads (Beckman Coulter, Brea, CA, USA) were applied to purify the obtained PCR amplicons. The Agilent 2100 Bioanalyzer (Agilent Technologies, Santa Clara, CA, USA) was used to evaluate library fragment size distribution, and real-time PCR was adopted for library quantification. Valid DNA libraries were sequenced on the Illumina HiSeq 1000 (Illumina, San Diego, CA, USA) platform at Beijing Novogene Bioinformatics Technology Co., Ltd., Beijing, China. Trimmomatic v0.39 [28], FastQC (https://www.bioinformatics.babraham.ac.uk/projects/fastqc) (accessed on 14 August 2023), SPAdes v3.14 [29], and Prokka v1.14.5 [30] were used for quality control, assembly, and annotation.

One of the 59 *Salmonella* isolates harboring the *mcr-9.2* gene was further subjected to whole-genome sequencing (WGS) performed by Shanghai Majorbio Bio-pharm Technology Co., Ltd., Shanghai, China. The data generated by the second- (using the Illumina HiSeq 1000 platform) and third-generation sequencing technology (using the PacBio Sequle lle platform) (Pacific Biosciences, Menlo Park, CA, USA) were assembled and annotated by the online Majorbio Cloud Platform (www.majorbio.com) [31].

### 2.2. Bioinformatics and Phylogenetic Analysis

Serotyping and multilocus sequence typing (MLST) of the *Salmonella* assemblies were performed using SeqSero2 v1.2.1 [32] and mlst v2.19 (https://github.com/tseemann/mlst (accessed on 18 February 2026)), respectively. Antimicrobial resistance (AMR) and virulence genes in each *Salmonella* genome were identified with ABRicate v1.01 (https://github.com/tseemann/abricate (accessed on 18 February 2026)) using the ResFinder (https://genepi.food.dtu.dk/resfinder (accessed on 18 February 2026)) and VFDB (http://www.mgc.ac.cn/VFs/main.htm (accessed on 18 February 2026)) databases with 95% identity and 90% query coverage cutoffs. The mob_recon and mob_typer tools in MOB-suite v3.1.8 were used to identify and extract plasmid-derived contigs from the assemblies, with identity and coverage thresholds set at 85% and 60%, respectively [33]. The AMR genes located on these plasmid-derived contigs were also screened. For phylogenetic analysis, the *S*. Typhimurium LT2 reference genome (GenBank: AE006468.2) was used. Single nucleotide polymorphisms (SNPs) from all *Salmonella* genomes were extracted using Snippy v4.4.5 (https://github.com/tseemann/snippy (accessed on 18 February 2026)). Recombination within the core genome alignments was removed using Gubbins v2.4.1 [34]. A phylogenetic tree was constructed with the maximum likelihood method using FastTree v1.4.3 [35]. For the comparative genome analysis, the *mcr-9.2*-producing *Salmonella* assemblies were downloaded from the NCBI pathogen database (accessed on 25 December 2025, Supplementary Material Data Set S1). The plasmid-derived contigs harboring the *mcr-9.2* gene were extracted from these genomes by MOB-suite v3.1.8, followed by comparative and phylogenetic analysis.

### 2.3. Visualization and Statistical Analysis

An undirected graph was created using Gephi v0.10.1 (https://github.com/gephi/gephi (accessed on 18 February 2026)) to visualize the interconnected AMR genes and *Salmonella* serovars. Sankey diagram and an UpSet plot were created using an R script (v3.6.2) (https://www.r-project.org/) with the networkD3 package (https://cran.r-project.org/web/packages/networkD3/index.html (accessed on 18 February 2026)) and ComplexUpset package (https://cran-e.com/package/ComplexUpset (accessed on 18 February 2026)) to exhibit the serovars, plasmid replicon types, relaxase types, predicted mobility, AMR genes, and insertion sequences among resistant plasmids. The *mcr-9.2* genes were compared and visualized with Easyfig v2.2.2 [36] and BLAST Ring Image Generator (BRIG; http://brig.sourceforge.net/). The phylogenetic tree was visualized by iTOL (https://itol.embl.de (accessed on 18 February 2026)) [37] and Grape-Tree (https://github.com/achtman-lab/GrapeTree (accessed on 18 February 2026)) [38].

Statistical analysis of the AMR and virulence genes among different *Salmonella* serovars was conducted using SPSS (version 24.0, Armonk, NY, USA: IBM Corp.), and GraphPad Prism (version 8.0, GraphPad Software, San Diego, CA, USA) was employed for data visualization. The Kruskal–Wallis test was used for multiple-group comparisons, as the data did not meet the assumptions of normality and homogeneity of variance. Post hoc pairwise comparisons were performed using Dunn’s test with Bonferroni correction. A *p*-value < 0.05 was considered statistically significant.

## 3. Results and Discussion

### 3.1. Isolate Characteristics and Genome Landscape

**Figure 1:**
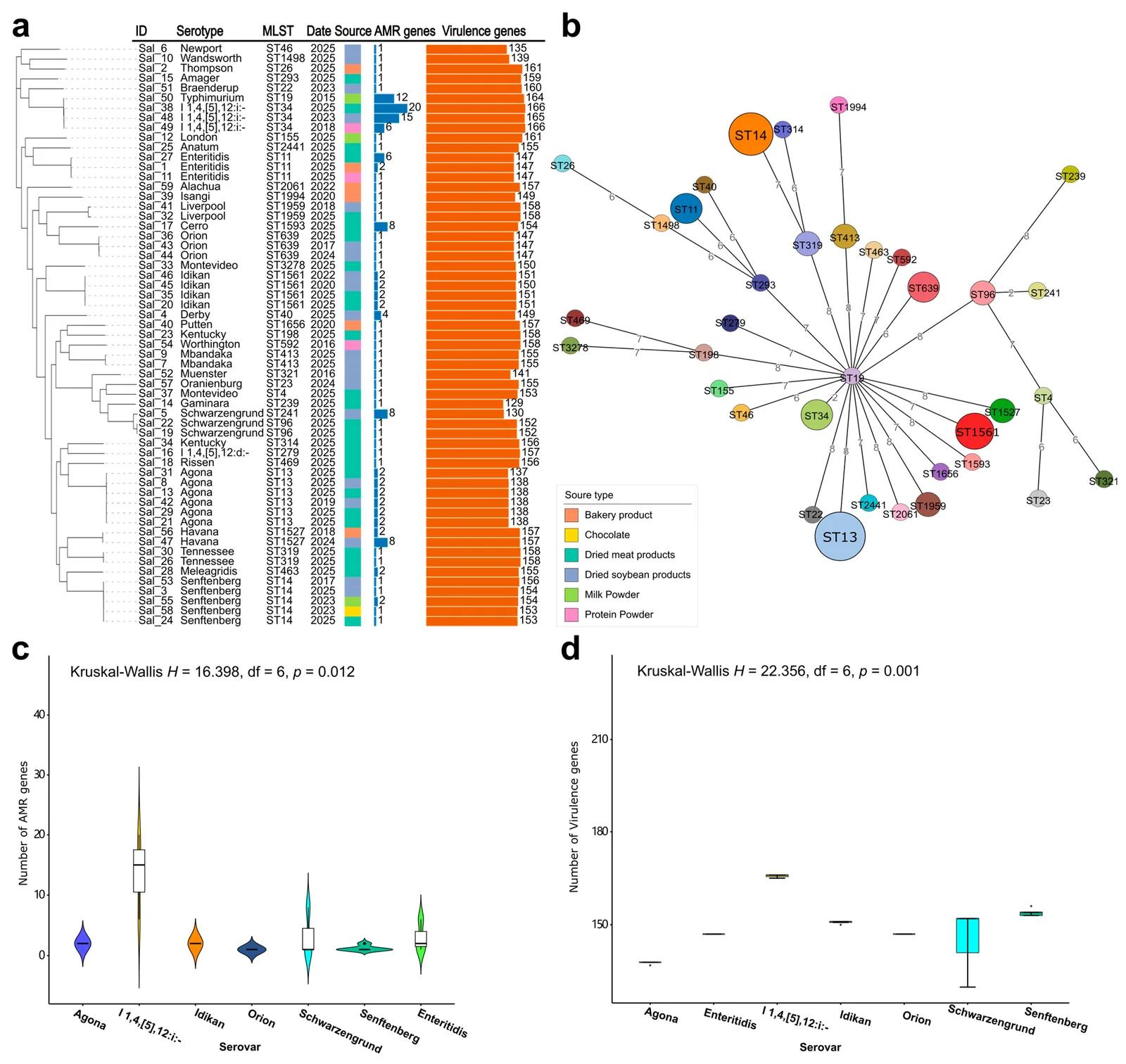
General features of 59 *Salmonella* strains from low-water-activity foods collected from 2017 to 2025 in Beijing, China. (**a**) Phylogenetic tree of 59 strains. From left to right: phylogenetic tree, serovars, sequence types, sampling date, food source types, number of resistance and virulence genes. (**b**) Minimum spanning tree of 59 strains. Sequence types were exhibited in different colors, with the number of strains of each sequence type represented by the node size. (**c**,**d**) The statistical differences in AMR and virulence genes among the top seven detected *Salmonella* serovars in this study.

A total of 59 *Salmonella* strains from low-water-activity foods were collected from 2017 to 2025 in Beijing, China (Figure 1 and Supplementary Table S1). In detail, a total of 38 *Salmonella* strains isolated from low-water-activity foods were collected in 2025, while 1 to 4 *Salmonella* strains were collected in other years, respectively (Figure 1a). Most strains were recovered from dried meat products (44.07%, 26/59), followed by 20 strains (33.90%, 20/59) from dried soybean products, six from bakery products (10.17%, 6/59), three of each (5.08%, 3/59) from milk powder and protein powder, and one from chocolate. The sub-typing results showed relatively high diversity, with a total of 33 serovars and 36 sequence types (STs) identified (Figure 1a,b). Among them, *S.* Agona (ST13) was the most prevalent (10.17%, 6/59), followed by *S.* Senftenberg (ST14) (8.47%, 5/59) and *S.* Idikan (ST1561) (6.78%, 4/59). Three of each (5.08%, 3/59) were identified as Enteritidis (ST11), I 1,4,[5],12:i:- (ST34), Orion (ST639), and Schwarzengrund (2 ST96 and 1 ST241), while two of each (3.39%, 2/59) were identified as Havana (ST1527), Kentucky (ST198 and ST314), Liverpool (ST1959), Mbandaka (ST413), Montevideo (ST4 and ST3278), and Tennessee (ST319). Each of the other serovars was represented by one strain (1.69%, 1/59).

Low-water-activity foods were traditionally regarded as microbiologically safe due to their inhibition of microbial growth. However, accumulating evidence has confirmed that *Salmonella* can survive for a long time in such foods and become an important hidden source of infection [22,25]. In this study, 59 *Salmonella* strains isolated from low-water-activity foods in Beijing showed high serotypic and genotypic diversity, covering 33 serovars and 36 STs, which reflected the complex contamination sources of *Salmonella* in low-water-activity foods in this region. Dried meat products and dried soybean products were the main contaminated food types, accounting for 44.07% and 33.90% of the total isolates, respectively. The dried meat products, such as dried shrimp, fish, and sausage, are highly susceptible to contamination by pathogens including *Salmonella* from raw materials or cross-contamination during processing and storage [39,40]. Similarly, dried soybean products could also be contaminated with *Salmonella* from processing to storage [41]. Of note, several foodborne outbreaks linked to these foods contaminated with *Salmonella* have been reported [42,43], suggesting that animal-derived and plant-derived dehydrated products are key monitoring objects for *Salmonella* control in low-water-activity foods. These results indicate that low-water-activity foods cannot be ignored in food safety risk assessment, and targeted monitoring measures need to be strengthened.

### 3.2. Diversity and Distribution of Antimicrobial Resistance and Virulence Genes

**Figure 2:**
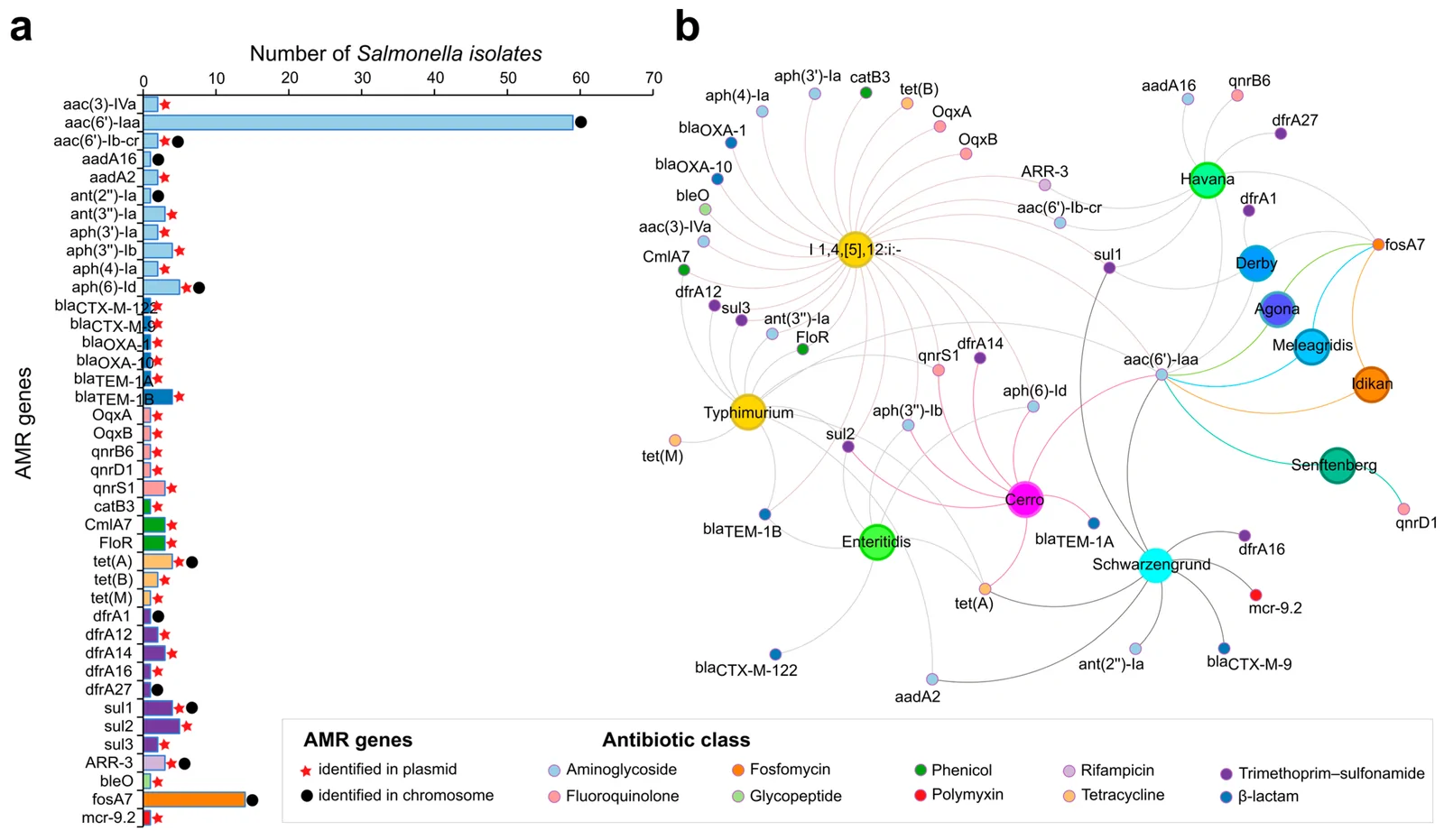
Distribution of AMR genes among chromosomes and plasmids (**a**) and undirected network connecting AMR genes to major serovars (**b**). The bar chart and AMR gene nodes are color-coded by their conferring antibiotic classes, while the nodes of different serovars are also displayed in distinct colors.

A total of 40 AMR genes spanning 10 classes were identified across the 59 *Salmonella* genomes (Figure 1 and Figure 2, and Table 1). The most prevalent AMR genes were *aac(6′)-Iaa* (*n* = 59), *fosA7* (*n* = 14), *aph(6)-Id* (*n* = 5), and *sul2* (*n* = 5) (Figure 2a and Table 1). A comparative analysis of the AMR genes among the top seven detected *Salmonella* serovars was performed. Twenty different AMR genes were identified in monophasic variant of *Salmonella enterica* subsp. *enterica* serovar Typhimurium (1,4,[5],12:i:-). These strains were found to harbor an average of 14 AMR genes (Figure 2b). This was significantly higher than those of other serovars, which carried an average of ≤3 AMR genes (Kruskal–Wallis H = 16.398, df = 6, *p* = 0.012) (Figure 1c). This pronounced disparity points to an enhanced propensity of monophasic variant of *Salmonella enterica* subsp. *enterica* serovar Typhimurium (1,4,[5],12:i:-) for resistance gene accumulation, which may confer superior environmental fitness and a competitive advantage within low-water-activity food matrices.

Whole-genome sequencing was utilized to profile antimicrobial resistance determinants across isolates. Consistent with earlier *Salmonella* surveillance work, *aac(6′)-Iaa*, an aminoglycoside-resistant determinant, was broadly distributed within our dataset, while *fosA7* represented the dominant fosfomycin-resistant gene. Aminoglycoside resistance commonly arises via enzymatic modification driven by phosphotransferase, acetyltransferase, and adenyltransferase proteins encoded by diverse resistance loci [44,45]. Ten *fosA* allelic variants have been characterized among Enterobacteriaceae, starting from the initial identification of *fosA1* in *Serratia marcescens* [46,47]. So far, *fosA7* has been detected globally in various *Salmonella* serovars [48,49,50]. Globally, *fosA7* occurs across multiple *Salmonella* serovars, and its widespread occurrence raises One Health-relevant public-health concerns [51]. Viewed through a One Health lens, these observations on fosfomycin resistance in *Salmonella* are deeply concerning and underscore the need for sustained surveillance.

β-Lactam resistance is mediated by bla genes encoding enzymes that hydrolyze the β-lactam ring. In the present cohort, *bla* genes were distributed across major serovars, comprising monophasic variant of *Salmonella enterica* subsp. *enterica* serovar Typhimurium (1,4,[5],12:i:-) (*bla*_OXA-1_, *bla*_OXA-10_, *bla*_TEM-1B_), *S*. Typhimurium (*bla*_TEM-1B_), *S*. Enteritidis (*bla*_CTX-M-122_, *bla*_TEM-1B_), and *S*. Schwarzengrund (*bla*_CTX-M-9_). blaTEM-1B was recurrently identified and has been previously associated predominantly with ampicillin-resistant *Salmonella* serovars [52]. Additional *tet* genes [*tet(A)* and *tet(B)*] and chloramphenicol resistance genes (*cmlA7* and *floR*) were also detected. Resistance to tetracyclines and chloramphenicol is often associated with efflux pump mechanisms encoded by *tet* and *floR* genes, respectively [53]. Notably, strains of the monophasic variant of *Salmonella enterica* subsp. *enterica* serovar Typhimurium (1,4,[5],12:i:-) carried significantly more antimicrobial resistance and virulence genes than other serovars, suggesting that this serovar had stronger environmental adaptability and pathogenic potential and that it spread in low-water-activity foods, which deserved close attention.

**Figure 3:**
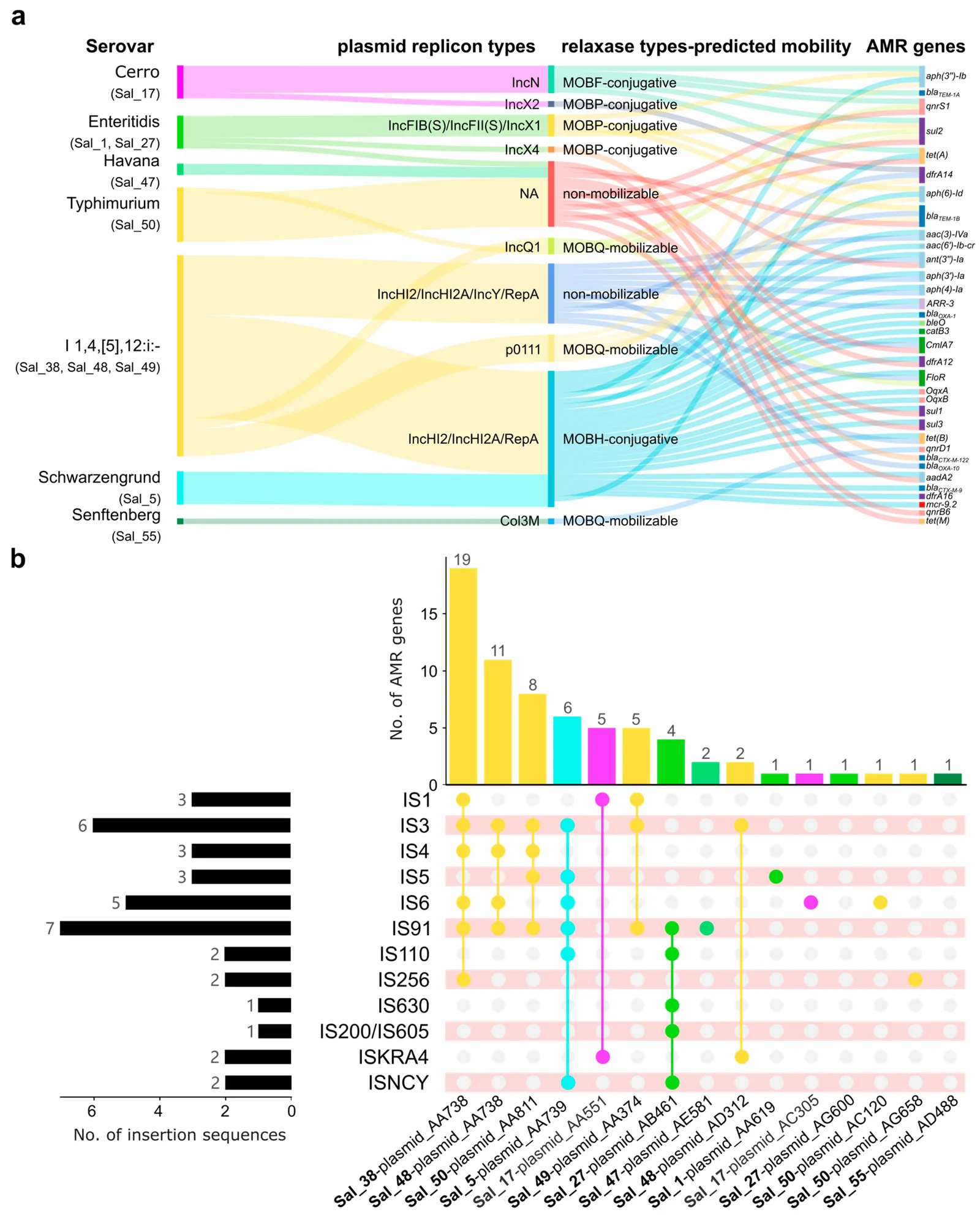
Sankey diagram exhibiting serovars’ plasmid replicon types, predicted mobility, and AMR genes (**a**). The Upset plot illustrating the co-occurrence of AMR genes and insertion sequences (IS) (**b**) among 15 resistant plasmids. (**a**) The width of each segment is proportional to the number of AMR genes within each category, highlighting the co-occurrence patterns of these mobile genetic elements. The text in parentheses below each serovar indicates the isolate IDs of the strains carrying the corresponding resistant plasmids. The serovars, plasmid replicon types, and AMR genes were color-coded. (**b**) The top bar graph represents the total number of AMR genes detected for each resistant plasmid. The bottom bar graph indicates the number of each insertion sequence. The matrix of colored dots in the middle shows the presence of each IS-plasmid pair, with colors corresponding to distinct serovars, revealing serovar-specific patterns of mobile genetic element co-occurrence.

Further analysis of AMR genes was performed on chromosome- and plasmid-derived contigs extracted from the genome using MOB-suite (Figure 3a). The results showed that only 15 types of plasmids carrying AMR genes were detected in 10 *Salmonella* strains. One strain of the serovars Cerro, Enteritidis, and the monophasic variant of *Salmonella enterica* subsp. *enterica* serovar Typhimurium (1,4,[5],12:i:-) possessed two resistant plasmids, while one *S*. Typhimurium strain harbored three resistant plasmids. A total of 34 AMR genes were identified in these resistant plasmids, among which 29 were exclusively present on the resistant plasmids, and the other five genes were detected in both plasmids and chromosomal sequences (Figure 2a). In addition, six AMR genes were uniquely detected in chromosomal sequences.

Among the 15 resistance plasmids, 11 could be assigned identifiable replicon types (Figure 3a). Four plasmids exhibited multiple replicon types, predominantly IncHI2/IncHI2A, IncFIB(S)/IncFII(S)/IncX1, and IncHI2/IncHI2A/RepA configurations (Table 2). Two plasmids recovered from isolates of the monophasic variant of *Salmonella enterica* subsp. *enterica* serovar Typhimurium (1,4,[5],12:i:-) and one plasmid from *S*. Typhimurium carried abundant AMR genes (19, 11, and 8 AMR genes, respectively), although all three were predicted to be non-mobilizable (Figure 3b). In contrast, eight other plasmids—carrying between one and six resistance genes—were predicted to be conjugative or mobilizable. A comprehensive summary of plasmid features, including replicon types, MOB types, AMR gene carriage, and insertion sequences, is provided in Table 2.

The insertion sequences (IS) were identified from 13 out of these 15 resistant plasmids, with IS*91* (*n* = 7), IS*3* (*n* = 6), and IS*5* (*n* = 5) being the top three frequently identified (Figure 3b). The conjugative plasmids with MOB type AA739 in one *S*. Schwarzengrund strain and AB461 in one *S*. Enteritidis strain possessed 6 and 5 insertion sequences, respectively, with the other conjugative and mobilizable plasmids possessing 0 to 3 insertion sequences. Moreover, the top three AMR genes harboring non-mobilizable plasmids, two AA738 plasmids and one AA811 plasmid, were found to possess four to six insertion sequences.

Mobile genetic elements (MGEs) constitute primary drivers of AMR dissemination by facilitating resistance gene transfer between bacterial cells [54]. In this study, we identified 15 different plasmids carrying AMR genes from 10 strains, among which the IncHI2/IncHI2A plasmids were significantly associated with MDR. IncHI2 plasmids rank among the most prevalent plasmid types in Enterobacteriaceae [55]. These are large (>250 kb) conjugative elements with broad host ranges, capable of shuttling both metal and drug resistance genes among Gram-negative pathogens [56]. Moreover, one plasmid of the *S*. Schwarzengrund strain that belongs to the IncHI2 incompatibility group was found to carry the *mcr-9.2* gene. IncHI2 type plasmids carrying the *mcr-9* gene have recently been characterized as “super plasmids” closely linked to diverse antimicrobial resistance determinants [57]. These predominant insertion-sequence families (IS91, IS3, and IS5) can facilitate plasmid recombination and acquisition of resistance cassettes, thereby accelerating the evolution and dissemination of drug-resistant bacterial isolates [58,59]. Although some resistance plasmids were predicted to be non-mobilizable, their stable existence in *Salmonella* still meant a persistent risk of resistance accumulation. The comparative results of the virulence genes among the top detected seven *Salmonella* serovars also showed significant differences among different serovars (Kruskal–Wallis H = 22.356, df = 6, *p* = 0.001) (Figure 1d). Of note, strains of the monophasic variant of *Salmonella enterica* subsp. *enterica* serovar Typhimurium (1,4,[5],12:i:-) possessed the highest number of virulence genes, with an average of 166, covering virulence-factor categories of adherence, invasion, effector delivery system, exotoxin, immune modulation, nutritional metabolic factor, competitive advantage-related determinants, and regulation (Supplementary Table S1).

### 3.3. The mcr-9.2-Producing IncHI2 Plasmid in Salmonella Schwarzengrund and the Genetic Environment Surrounding the mcr-9.2 Gene

**Figure 4:**
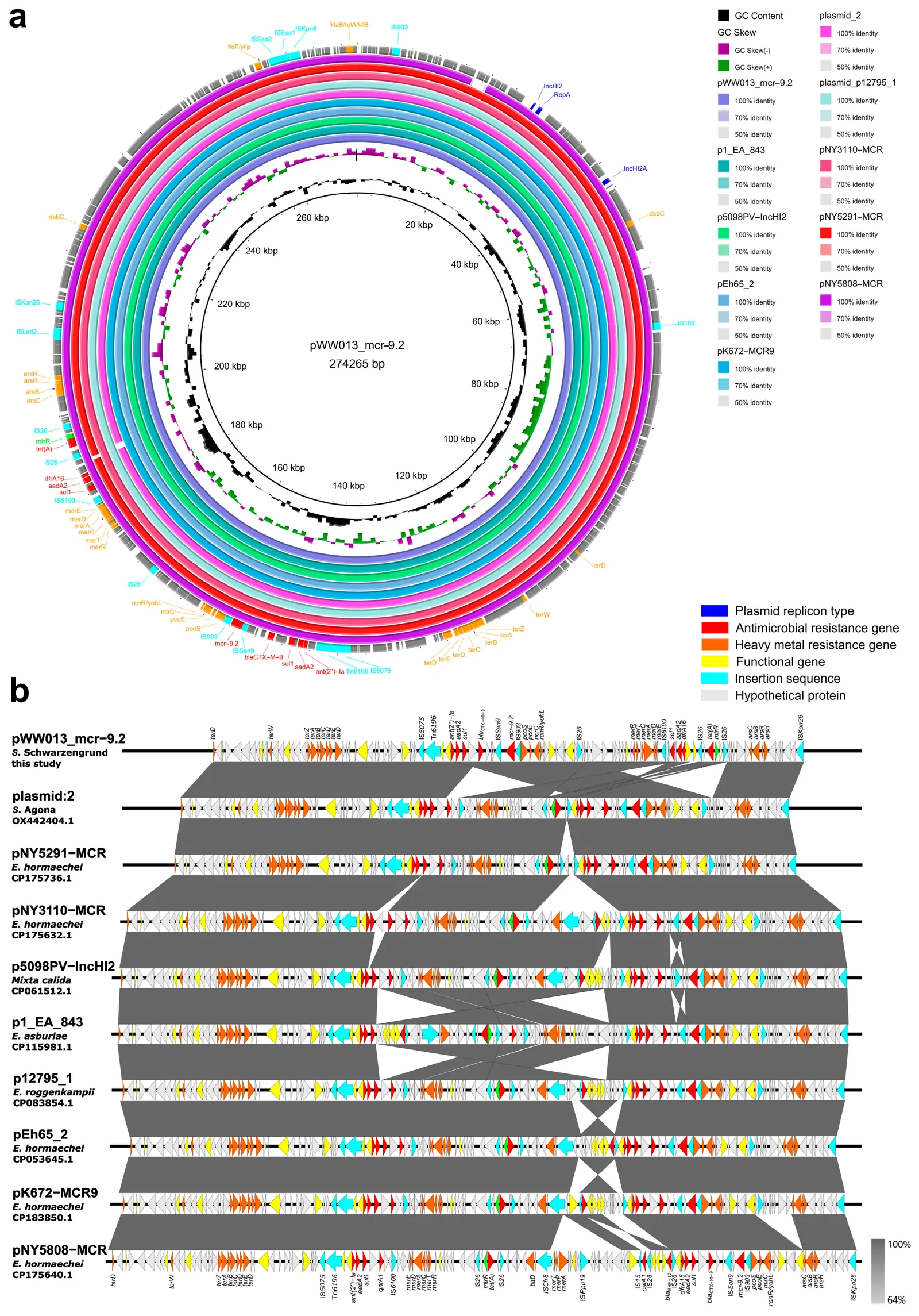
Alignment of *mcr-9.2*- positive IncHI2 plasmid and Linear comparative analysis surrounding the *mcr-9.2* gene identified in one *Salmonella* Schwarzengrund strain in this study and those in the NCBI database. (**a**) Circular map of pWW013_mcr-9.2 (274,265 bp). The circles from the center outward show GC skew (green/purple), GC content (black), and sequence similarity to nine reference plasmids. Key functional elements are annotated: antimicrobial resistance genes (red), heavy metal resistance genes (orange), insertion sequences (cyan), and plasmid replicon type (blue). (**b**) Linear comparative analysis of pWW013_mcr-9.2 with nine closely related IncHI2 plasmids from Enterobacteriaceae species. Gray blocks represent regions of nucleotide identity. Functional genes are annotated with colored arrows. Detailed information, including GenBank accession numbers for the nine reference plasmids in panel (**b**), is provided in Table 3.

One *S*. Schwarzengrund strain from the dried soybean product harboring plasmid pWW013_mcr-9.2 was identified in this study. The plasmid is a circular DNA molecule of 274,265 bp with an average GC content of 46.2% and belongs to the IncHI2 incompatibility group (Figure 4a). The backbone regions, including genes for replication, maintenance, and conjugative transfer, were largely conserved and characteristic of IncHI2 plasmids. Visualization of the plasmid using BRIG revealed that accessory elements, including resistance genes and insertion sequences, were concentrated in several discrete genomic islands, while GC skew analysis indicated the presence of multiple recombination hotspots (Figure 4a). In silico resistance gene analysis identified multiple AMR determinants in pWW013_mcr-9.2. Notably, the plasmid carried the *mcr-9.2* gene. Additional AMR genes were detected, including those conferring resistance to β-lactams (*bla*_CTX-M-9_), aminoglycosides [*aadA2* and *ant(2″)-Ia*], trimethoprim-sulfonamides (*dfrA16* and *sul1*), and tetracycline [*tet(A)*]. Heavy metal resistance operons, including those for mercury (*mer*), arsenic (*ars*), and tellurium (*ter*), were also present. Insertion sequences were abundant throughout the plasmid, particularly in the vicinity of resistance genes (Figure 4a). The *mcr 9* family represents widely distributed genetic determinants associated with colistin resistance [60]. Distinct from other *mcr* variants, *mcr 9* does not consistently produce constitutive phenotypic resistance; its capacity for silent expression enables cryptic circulation and raises substantial public health concerns [15,60,61,62].

**Table 3:** Metadata of nine reference plasmids in Figure 4b.

Plasmid ID	GenBank Accession No.	Host Organism
plasmid:2	OX442404.1	*S.* Agona
pNY5291-MCR	CP175736.1	*E. hormaechei*
pNY3110-MCR	CP175632.1	*E. hormaechei*
p5098PV-IncHI2	CP061512.1	Mixta calida
p1_EA_843	CP115981.1	*E. asburiae*
p12795_1	CP083854.1	*E. roggenkampii*
pEh65_2	CP053645.1	*E. hormaechei*
pK672-MCR9	CP183850.1	*E. hormaechei*
Pny5808-MCR	CP175640.1	*E. hormaechei*

To explore the genetic relatedness and evolutionary context of pWW013_mcr-9.2, we performed comparative genomic analysis with nine closely related IncHI2 plasmids carrying *mcr-9.2* genes, retrieved from the NCBI GenBank database (Figure 4b). Linear alignment using Easyfig demonstrated that the plasmid backbone was highly conserved across all compared plasmids, with nucleotide identities exceeding 90%. In contrast, extensive structural variations were observed in the accessory regions, particularly within the MDR islands. The *mcr-9.2*-containing locus in pWW013_mcr-9.2 exhibited a unique genetic arrangement that differed from other *mcr-9.2*-carrying IncHI2 plasmids, including pNY5291-MCR, pNY3110-MCR, and p5098PV-IncHI2. Some reference plasmids, such as p1_EA_843 and p12795_1, showed large deletions or rearrangements in the resistance regions.

Our data also showed that this *mcr-9.2*-carrying IncHI2/IncHI2A plasmid carried *bla*_CTX-M-9_, *aadA2*, *ant(2″)-Ia*, *dfrA16*, *sul1*, *tet(A),* and other resistance genes, as well as mercury, arsenic, tellurium, and other heavy metal resistance operons, forming a complex multidrug and heavy metal co-resistance region. Previous studies have reported that plasmids carry both heavy metal and AMR genes, indicating a potential for co-selection through co-resistance [63,64,65]. Co-selection pressure exerted by heavy metals contributes to the dissemination and persistence of AMR genes in environmental reservoirs and facilitates the stable maintenance and spread of these plasmids [65]. Comparative genomic analysis showed that the backbone of pWW013_*mcr-9.2* was highly conserved with other *mcr-9.2*-carrying IncHI2 plasmids, but the structure of the resistance island was unique, indicating the local evolutionary characteristics of *mcr-9.2* plasmids in China. Our findings further provide evidence for the above theory, and such plasmids deserve close attention in future research.

### 3.4. Global Prevalence and Epidemiological Traits of mcr-9.2-Positive Plasmids Among Salmonella Assemblies in the NCBI Database

**Figure 5:**
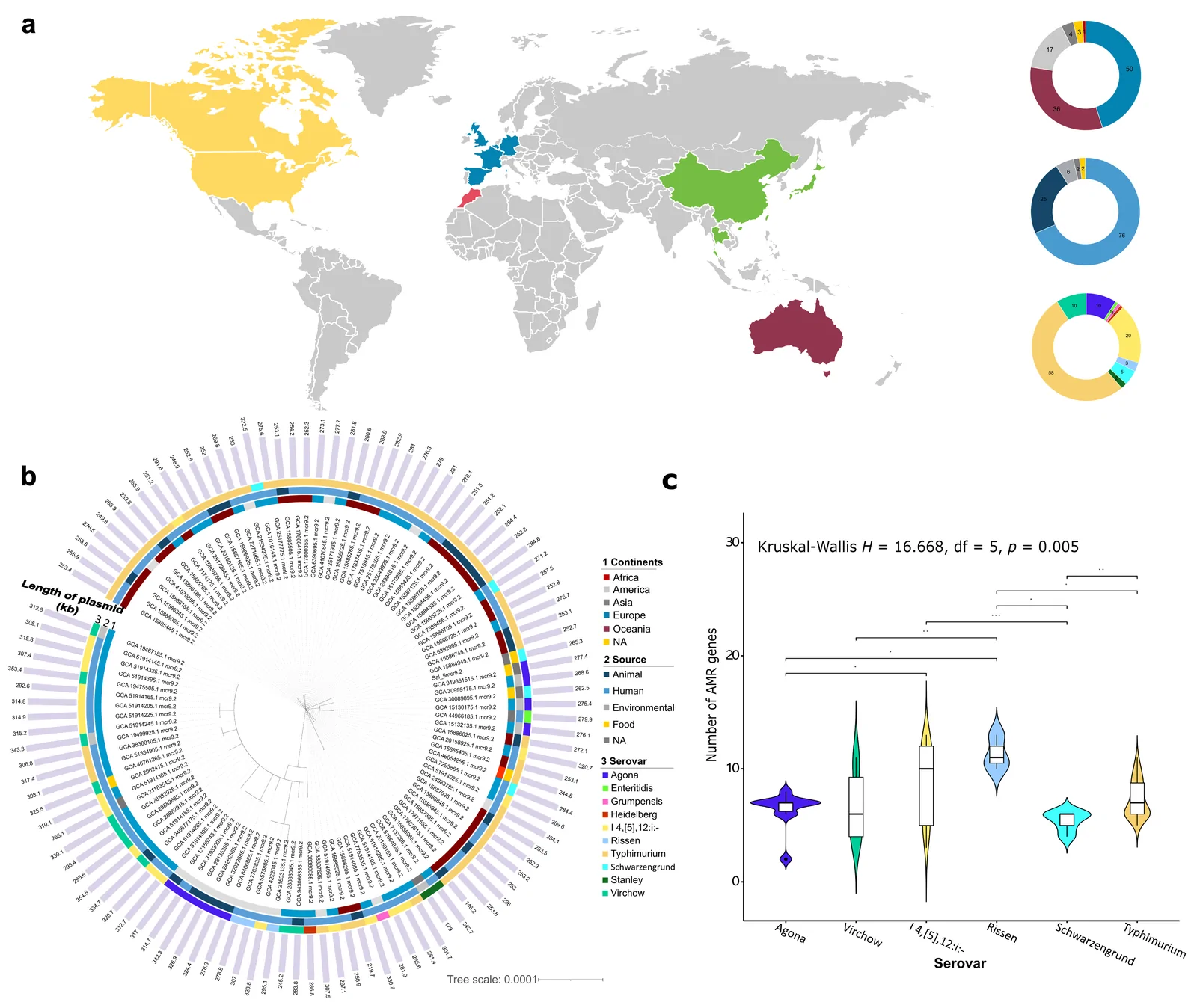
Global distribution map of *mcr-9.2*-carrying *Salmonella* found in this study and the NCBI database (**a**), phylogenetic tree of plasmid-harboring *mcr-9.2* (**b**), and relationship between the number of antimicrobial resistance genes among serovars (**c**). The pie charts show the distribution of the *mcr-9.2*-carrying *Salmonella* continents, sources, and serovars.

A total of 110 publicly available *Salmonella* carrying *mcr-9.2* were retrieved from the NCBI GenBank database. All these *mcr-9.2*-positive plasmids were identified as IncHI2/IncHI2A plasmids (Supplementary Material Data Set S1). Figure 5a depicts the prevalence and geographical distribution of these assemblies, including the *mcr-9.2*-positive *S*. Schwarzengrund strain in this study. Only one assembly was from Africa. The majority were derived from economically developed regions. In terms of isolation source, most were of human origin with 76 assemblies, followed by smaller fractions derived from animals (25 assemblies), environmental sources (six assemblies), and food (two assemblies). Serovar analysis revealed that the plasmids were primarily associated with *S.* Typhimurium (58 assemblies) and the monophasic variant of *Salmonella enterica* subsp. *enterica* serovar Typhimurium (1,4,[5],12:i:-) (20 assemblies), Virchow (10 assemblies), *S*. Agona (10 assemblies), and *S*. Schwarzengrund (five assemblies), with additional isolates belonging to other serovars. The results demonstrated considerable sequence diversity among the IncHI2/IncHI2A plasmids (Figure 1b). Plasmid length varied widely, ranging from 146,159 bp to 354,517 bp, with an average of 279,896 bp. Global epidemiological analysis of *mcr-9.2*-positive *Salmonella* showed that most isolates were from Europe (*n* = 50) and Oceania (*n* = 36), predominantly from human clinical specimens, with fewer isolates from the Americas (*n* = 17) and Asia (*n* = 4), and only one from Africa. The detection of this *mcr-9.2*-carrying *S*. Schwarzengrund strain in the current study suggested that *mcr-9.2* was in an emerging and under-monitored state in Asia [61]. Moreover, previous reports also revealed that serovars Typhimurium and the monophasic variant of *Salmonella enterica* subsp. *enterica* serovar Typhimurium (1,4,[5],12:i:-) were the dominant serovars carrying *mcr-9.2* globally [61]. Only four *mcr-9.2*-positive *S*. Schwarzengrund genomes are publicly available in NCBI (all from Europe), which made the strain found in this study a potentially novel epidemiological sentinel for the region. The detection of *mcr-9.2*-positive *Salmonella* from low-water-activity food in this study indicates that an *mcr-9.2*-carrying plasmid has been detected in the food chain beyond the clinical setting. Due to the inducible expression characteristics of *mcr-9.2*, conventional phenotypic detection is easy to miss positive strains, which highlights the important value of whole-genome sequencing in antimicrobial resistance surveillance.

The number of AMR genes harbored by the *mcr-9.2*-positive plasmids varied significantly among different *Salmonella* serovars (Figure 1c). Plasmids from serovar Rissen carried the highest number of AMR genes (average = 11), followed by the monophasic variant of *Salmonella enterica* subsp. *enterica* serovar Typhimurium (1,4,[5],12:i:-) (average = 9), and Typhimurium (average = 7). In contrast, plasmids from serovar Schwarzengrund carried the lowest median number of AMR genes (average = 5). Statistical analysis using the Kruskal–Wallis test confirmed these differences were significant (H = 16.668, df = 5, *p* = 0.005). Post hoc comparisons revealed that plasmids from Rissen carried significantly more AMR genes than those from Agona, Virchow, Schwarzengrund, and Typhimurium (*p* < 0.05). IncHI2 plasmids are particularly worrisome by virtue of their large size, broad host spectrum, abundant MGE payload, and conjugative transfer competence, all of which enable them to serve as backbones for complex resistance islands harboring antibiotic resistance genes, heavy metal tolerance loci, and biocide-resistant determinants [66,67,68]. These findings underscore notable serovar-dependent differences in the resistomes of *mcr-9.2*-carrying plasmids, with meaningful implications for public health risk stratification.

The global phylogenetic analysis of *mcr-9.2*-positive plasmids among *Salmonella* showed that plasmids from different continents, sources, and serovars were interspersed within the tree, suggesting the potential for cross-regional and cross-host dissemination of these *mcr-9.2*-harboring elements. The overall plasmid length ranged from 200 kb to over 300 kb, consistent with the size of typical IncHI2 plasmids [56]. The IncHI2 plasmids are particularly concerning among plasmid incompatibility groups. It has been reported that IncHI2 plasmids serve as the transmission vector for various AMR genes [66]. These collective observations reinforce the imperative for sustained global surveillance of IncHI2 plasmids carrying *mcr-9.2* within the food supply chain.

## 4. Limitations

The present study has several noteworthy limitations. First, isolate sampling was restricted to Beijing with a limited sample size. The dataset presents a heavily skewed temporal distribution: 38 out of 59 isolates (64%) were obtained from targeted surveillance in 2025, whereas only 14 isolates were recovered per year for the remaining years. Given this uneven sampling across years, temporal trends in serovar and antimicrobial resistance prevalence cannot be reliably inferred, and observed patterns cannot be generalized to nationwide *Salmonella* populations from low-water-activity foods. Second, all findings rely solely on whole-genome-based predictions, without phenotypic validation including colistin susceptibility testing and plasmid conjugation assays. Notably, *mcr-9* may be silenced or inducibly expressed, meaning the detection of *mcr-9.2* at the genomic level does not guarantee a colistin-resistant phenotype. Third, certain serovar subgroups contained only a limited number of isolates (*n* = 2–3 isolates). Such imbalanced sample sizes can lower the statistical power of the Kruskal–Wallis test and elevate the probability of both Type I and Type II statistical errors. For this reason, any significant *p*-values obtained under this unstable grouping framework need to be interpreted prudently. Future work should expand sampling coverage and incorporate phenotypic assays to trace transmission routes of drug-resistant *Salmonella* in food supply chains.

## 5. Conclusions

In summary, *Salmonella* isolates from low-water-activity foods exhibited high genomic diversity. Antimicrobial resistance genes were prevalent, although only a minority of isolates carried multiple determinants. The identified *mcr-9.2*-carrying IncHI2 plasmid highlights the emergence of a plasmid-borne *mcr-9.2* gene in the food chain. These findings provide genomic evidence for risk assessment and targeted surveillance of *Salmonella* in low-water-activity foods.

## Figures and Tables

**Table 1:** Distribution of 40 antimicrobial-resistant genes among 59 *Salmonella* isolates from low-water-activity foods.

AMR Gene	Number of Carrier Isolates	AMR Gene	Number of Carrier Isolates
*aac(3)-IVa*	2	*CmlA7*	3
*aac(6′)-Iaa*	59	*dfrA1*	1
*aac(6′)-Ib-cr*	2	*dfrA12*	1
*aadA16*	1	*dfrA14*	3
*aadA2*	2	*dfrA16*	1
*ant(2* *″* *)-Ia*	1	*dfrA27*	1
*ant(3* *″* *)-Ia*	3	*FloR*	3
*aph(3′)-Ia*	2	*fosA7*	14
*aph(3* *″* *)-Ib*	4	*mcr-9.2*	1
*aph(4)-Ia*	2	*OqxA*	1
*aph(6)-Id*	5	*OqxB*	1
*ARR-3*	3	*qnrB6*	1
*bla*_CTX-M-122_	1	*qnrD1*	1
*bla*_CTX-M-9_	1	*qnrS1*	3
*bla*_OXA-1_	1	*sul1*	4
*bla*_OXA-1__0_	1	*Sul* *2*	5
*bla*_TEM-1A_	1	*sul3*	2
*bla*_TEM-1B_	4	*tet(A)*	4
*bleO*	1	*tet(B)*	2
*catB3*	1	*tet(* *M* *)*	1

Note: These counts of antimicrobial-resistant genes were derived from the binary genotype matrix provided in Supplementary Table S1. The data correspond to the gene profiles visualized in Figure 2a.

**Table 2:** Characteristics of the 15 resistance plasmids identified in this study.

Plasmid ID	Serovar/Isolate	Replicon Type(s)	MOB/Mobility Class	No. AMR Genes	No. IS Elements
plasmid_AA738	*S*. I 1,4,[5],12:i:-	IncHI2/IncHI2A/RepA	non-mobilizable	19	7
plasmid_AA738	*S*. I 1,4,[5],12:i:-	IncHI2/IncHI2A/IncY/RepA	non-mobilizable	11	6
plasmid_AA811	*S*. Typhimurium	NA	non-mobilizable	8	4
plasmid_AA739	*S*. Schwarzengrund	IncHI2/IncHI2A/RepA	MOBH (conjugative)	6	6
plasmid_AA551	*S*. Cerro	IncN	MOBF (conjugative)	5	3
plasmid_AA374	*S*. I 1,4,[5],12:i:-	p0111	MOBH (mobilizable)	5	2
plasmid_AB461	*S*. Enteritidis	IncFIB(S)/IncFII(S)/IncX1	MOBP (conjugative)	4	5
plasmid_AE581	*S*. Havana	NA	non-mobilizable	2	1
plasmid_AD312	*S*. I 1,4,[5],12:i:-	IncQ1	non-mobilizable	2	2
plasmid_AD488	*S*. Senftenberg	Col3M	MOBQ (mobilizable)	1	1
plasmid_AA619	*S*. Enteritidis	IncX4	MOBP (conjugative)	1	2
plasmid_AC305	*S*. Cerro	IncX2	MOBP (conjugative)	1	2
plasmid_AG600	*S*. Enteritidis	NA	non-mobilizable	1	—
plasmid_AC120	*S*. Typhimurium	IncQ1	MOBQ (mobilizable)	1	1
plasmid_AG658	*S*. Typhimurium	NA	non-mobilizable	1	—

Note: NA, not assigned; —, not detected.

## Data Availability

The data presented in this study are openly available in NGDC Genome Sequence Archive (GSA) at https://ngdc.cncb.ac.cn/gsa/ (accessed on 18 February 2026), reference number CRA043793. All accession numbers of the publicly available genomes were listed in Supplementary Table S1 and Data Set S1. All reference genomes used in this study were retrieved from NCBI, all of which were publicly available and unrestricted for reuse.

## Supplementary Materials

The following supporting information can be downloaded at https://www.mdpi.com/article/10.3390/foods15183248/s1. Supplementary Table S1. Summary of the isolation, AMR and genomic characteristics of *Salmonella* isolates collected from low-water-activity foods in Beijing, China during 2017–2025. Data Set S1. Summary of 110 publicly available *mcr-9.2*-carrying *Salmonella* genomes retrieved from the NCBI GenBank database.
